# Changing COVID-19 cases and deaths detection in Florida

**DOI:** 10.1371/journal.pone.0299143

**Published:** 2024-03-28

**Authors:** Kok Ben Toh, Derek A. T. Cummings, Ira M. Longini, Thomas J. Hladish

**Affiliations:** 1 Department of Biology, University of Florida, Gainesville, Florida, United States of America; 2 Institute of Global Health and Department of Preventive Medicine, Northwestern University, Chicago, Illinois, United States of America; 3 Emerging Pathogens Institute, University of Florida, Gainesville, Florida, United States of America; 4 Department of Biostatistics, University of Florida, Gainesville, Florida, United States of America; The University of Hong Kong School of Public Health, HONG KONG

## Abstract

Epidemic data are often difficult to interpret due to inconsistent detection and reporting. As these data are critically relied upon to inform policy and epidemic projections, understanding reporting trends is similarly important. Early reporting of the COVID-19 pandemic in particular is complicated, due to changing diagnostic and testing protocols. An internal audit by the State of Florida, USA found numerous specific examples of irregularities in COVID-19 case and death reports. Using case, hospitalization, and death data from the the first year of the COVID-19 pandemic in Florida, we present approaches that can be used to identify the timing, direction, and magnitude of some reporting changes. Specifically, by establishing a baseline of detection probabilities from the first (spring) wave, we show that transmission trends among all age groups were similar, with the exception of the second summer wave, when younger people became infected earlier than seniors, by approximately 2 weeks. We also found a substantial drop in case-fatality risk (CFR) among all age groups over the three waves during the first year of the pandemic, with the most drastic changes seen in the 0 to 39 age group. The CFR trends provide useful insights into infection detection that would not be possible by relying on the number of tests alone. During the third wave, for which we have reliable hospitalization data, the CFR was remarkably stable across all age groups. In contrast, the hospitalization-to-case ratio varied inversely with cases while the death-to-hospitalization ratio varied proportionally. Although specific trends are likely to vary between locales, the approaches we present here offer a generic way to understand the substantial changes that occurred in the relationships among the key epidemic indicators.

## Introduction

The first confirmed SARS-CoV-2 cases in Florida, USA, were reported in Mar 2020. By the end of 2022, more than 7 million infections were reported, resulting in more than 80 thousand COVID-19 deaths [1]. Due to changing testing and reporting policies [2, 3], a straightforward interpretation of the epidemic data is difficult, however, and a direct comparison of case numbers reported during different periods of the pandemic is inappropriate. In particular, limited testing capacity and strict testing criteria during the early stages of the pandemic likely systematically reduced the detection of asymptomatic and mild infections. In the State of Florida, a 2022 internal audit found numerous examples of irregularities in COVID-19 data collection and reporting during the early months of the pandemic ([4]). Auditors found that case and death data were sometimes missing, crucial demographic details were not reported, routine quality checks did not occur, and clear data discrepancies were not reconciled, or were reconciled using *ad hoc* methods. Despite these challenges, a proper understanding of the epidemiology of COVID-19 during this period and its surveillance is important for future pandemic preparedness.

Useful inferences can be made about detection and reporting dynamics by examining case, hospitalization, and death data over the course of the pandemic. While most widely reported, cases tend to be unreliable indicators during the early months of the pandemic, as they are highly influenced by the access and availability of tests ([5, 6]). Nationwide, the United States Centers for Disease Control and Prevention (CDC) estimated that the infection detection rate was 13% from Feb 2020 to Sep 2020, but month-to-month variability was high ([7]). Triangulating the case numbers with hospitalizations and deaths—which are smaller numbers but perhaps more consistently reported—may help to uncover the details of how case detection changed over this period.

Hospitalization and death data, however, can have their own reporting challenges, particularly during the early stages of a pandemic. Collating data from hospitals can be challenging without adequate resources, with the set of reporting hospitals changing day-to-day. Deaths can be undercounted, as shown by the discrepancy between reported COVID-19 deaths and excess deaths during the pandemic ([8]).

In this study, we attempt to understand the overall epidemiology of COVID-19 in Florida during the first year of the pandemic by comparing all publicly available data on COVID-19 cases, hospitalizations and deaths. In addition to reported numbers by the State and CDC, we also use linelist data of reported cases that were publicly available from the beginning of the pandemic until early Jun 2021. This dataset was published by the Florida Department of Health (FDOH), updated daily, and included valuable information such as age group and whether the patient was hospitalized, or died. As these data are no longer available from the State, we have archived them and provide them via a Zenodo repository (https://zenodo.org/record/7921083). We verify the consistency of these data with other data sources and use it to investigate the changes in case and death detection among age groups over the first year of the pandemic.

## Materials and methods

### Data

The State linelist was publicly available through an ArcGIS REST API service from Mar 2020 to early Jun 2021, and was updated daily. In this study, we use the linelist downloaded on 26 May 2021. The data includes 2,316,142 detected case entries. For each entry, we used the following information: age, case reporting date, and whether the individual was hospitalized and/or eventually died. We use the linelist to calculate weekly new cases, hospital admissions, and deaths by the case reporting date.

The State also supplied daily reports of COVID-19 through its website ([9]), including cases, hospitalizations, and deaths until Jun 2021, when update frequencies became weekly. We used these daily reports to construct weekly cases, hospitalizations, and deaths by their reporting dates.

The CDC provided Florida’s daily new cases by case reporting date and daily new deaths by death date ([1]). We downloaded the dataset on 1 Aug 2022 for this study and constructed weekly cases by case reporting date, and weekly deaths by death date.

The United States Department of Health & Human Services (HHS) supplied the reported number of COVID-19 hospitalization daily ([10]). We downloaded this study’s dataset on 1 Aug 2022 and calculated the weekly COVID-19 hospital admission by admission date.

The CDC’s National Center of Health Statistics supplied Florida’s weekly all-cause mortality with age stratification from 2015 to 2022 ([11]). We downloaded the dataset on 1 Aug 2022 for this study and used the dataset to estimate excess deaths throughout the pandemic.

All datasets are or were publicly available, and we did not have any access to information that could identify individual cases recorded in any of these datasets. To our knowledge, the definition of cases, hospitalizations and deaths did not change over the study period.

### Study period and wave demarcation

Because the hospitalization and death status of a case in the state linelist was often not known until more than a month later, we end our analyses on 3 Apr 2021, roughly two months before the case records end in our linelist. Our study period is thus defined as 1 Mar 2020 to 3 Apr 2021.

During this period, Florida experienced three waves of COVID-19 transmission. We defined the first (spring) and second (summer) waves as weeks 10 to 19 (8 Mar to 9 May) and weeks 20 to 40 (10 May to 3 Oct) of 2020, i.e., from the week of lowest case incidence before a peak to the week of lowest incidence after the peak. Weeks are numbered according to CDC Morbidity and Mortality Weekly Report guidelines. The third (winter) wave is defined as week 41 (4 Oct) of 2020 through the end of the study period.

### Indicator normalization and confidence interval

To compare the trends among all indicators (cases, hospitalizations, and deaths) on the same scale, we first calculate the average weekly numbers during the first wave for each indicator. We then normalize each indicator by dividing each by that indicator’s average value in the first wave.

To account for observation or recording errors, we used a non-overlapping block-bootstrapping method on the time series [12, 13]. First, we apply the seasonal-trend decomposition to the time series [14]. The remainder were then sequentially split into 7-day blocks. For each bootstrapped time series, we randomly sampled the 7-day blocks with replacement and added the remainder to the seasonal and trend component. After constructing a bootstrapped time series, we conducted the normalization exercise. Using the 2000 bootstrapped time series, we then determined the 95% interval for the normalized indicators.

### Excess deaths

To estimate excess deaths, we first fit an ARIMA model to all-cause mortality data from 2015 to Feb 2020 (just before the pandemic began in Florida). ARIMA models are a common approach to forecast time series by describing the temporal autocorrelations in the data [15]. The strong temporal and seasonal trends in all-cause mortality allow us to construct the counterfactual, i.e., the number of mortalities had the COVID-19 pandemic not happened, using the ARIMA model. Based on the best cross-validation performance, we chose a model with a second-order autoregressive component and a first-order seasonal autoregressive and moving average component (52-week periodicity) with drift. We then used the model to predict the excess deaths on a weekly basis from Mar 2020 onward. Excess deaths were then calculated by subtracting the observed deaths from the modelled expected deaths. The model was fitted using R and the forecast package ([16]).

### Ethics statement

This study is approved as an IRB exempt study by the University of Florida IRB (IRB202200853). Since the data is publicly available and no personally identifiable information was included in the datasets, consent was not required.

## Results

### Cases and deaths, but not hospitalizations in the linelist are consistent with other state and federal sources

As a preliminary evaluation of the reliability of the Florida Department of Health (FDOH) COVID-19 linelist, we compared the case, hospitalization, and death information in the linelist with other data sources. These other sources include the FDOH daily COVID-19 reports, as well as the CDC and HHS.

**Fig 1:**
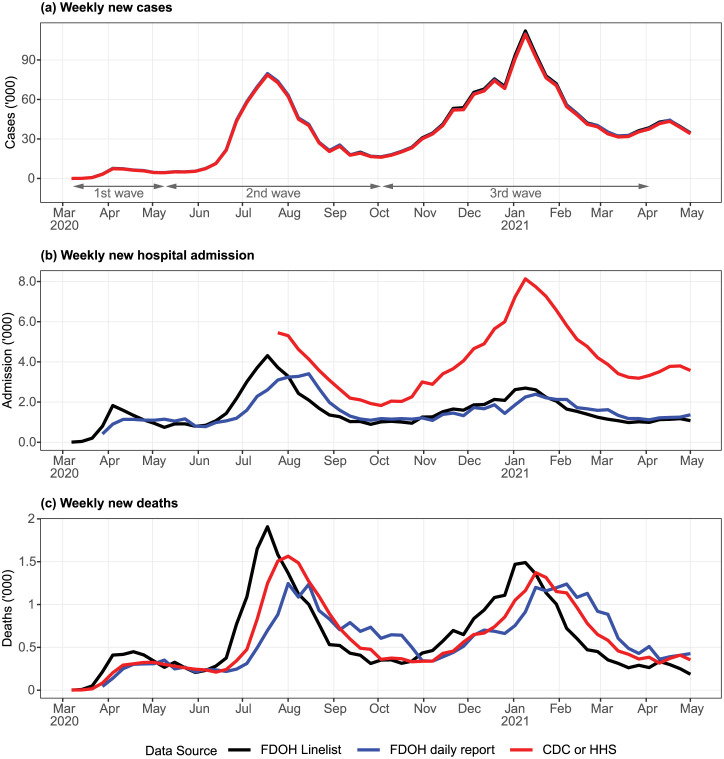
Weekly cases, hospitalizations, and deaths among different data sources. (a) All three case data sources are indexed using case reporting date. (b) Weekly hospital admissions were indexed by different dates: case reporting date for FDOH linelist, admission reporting date for FDOH daily report, and day of admission for HHS data. (c) Weekly deaths indexed by different dates: case reporting date for FDOH linelist, death reporting date for FDOH daily report, and day of death for CDC data.

Cases from all data sources are indexed by case reporting date and are near-identical (Fig 1a). In principle this could indicate that the data are reliable, but could also be because of a common, flawed source. In contrast, hospitalization estimates based on the State linelist and daily reports were likely underestimating the true number (Fig 1b). Hospital admission by case reporting date, derived from the State linelist, tended to lead the same metric by admission reporting date from the State’s daily report. This is not surprising, as cases could be detected prior to hospitalization for COVID-19. In terms of total number of hospital admissions, the two sources were consistent. However, the incidence of hospital admission based on the HHS data, which only became available in Jul 2020 when HHS mandated hospital reporting, was much higher. Since most of the hospitals in Florida reported to HHS regularly, we deem the HHS dataset as a more complete dataset. The sheer discrepancy between the State and HHS data, which worsened during the third wave, suggests that the State’s hospital admission numbers were incomplete. This is consistent with informal communications we have had with State health authorities.

Deaths reported from all data sources were largely consistent (Fig 1c), with the difference among the sources attributable to the different indexing dates. In general, deaths by case reporting date (based on FDOH linelist) led deaths by day of death (CDC data) by approximately 2 weeks. CDC data in turn led FDOH death reporting date, also by 2 weeks.

### Using the first wave as the baseline, cases and deaths increased at a different rate

To directly compare reporting trends in cases and deaths, we calculated the weekly cases and deaths by case reporting date, which can be done using the FDOH linelist. We normalized these time series with the mean weekly numbers of the corresponding indicator during the first wave. This allows us to track the growth rates of these indicators on a common scale. Because hospital admission data recorded in the linelist were inconsistent (as described in the previous section), we dropped the indicator from this analysis.

**Fig 2:**
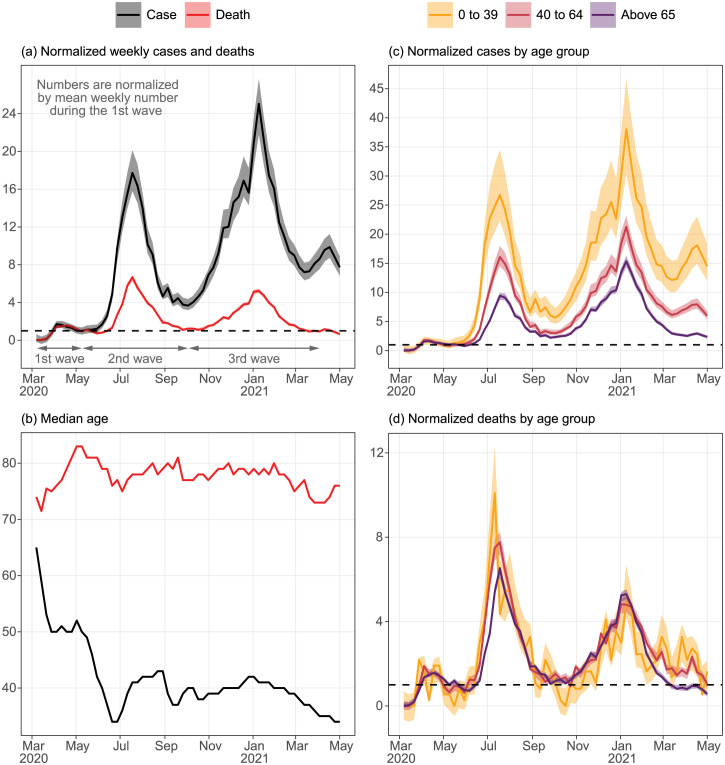
Population-wide and age-stratified normalized weekly COVID-19 cases and deaths and their weekly median age by case reporting date from Mar 2020 to Apr 2021. Cases and deaths are normalized by their corresponding mean weekly count during the first wave. All time series are derived from the State linelist and indexed according to the case reporting date. Dashed horizontal lines indicate a normalized count of 1. (a) Weekly normalized COVID-19 cases and deaths, (b) weekly median age of cases and deaths, and the age-stratified weekly normalized (c) cases and (d) deaths.

During the transition from the first to the second waves, cases grew more rapidly than deaths (Fig 2a). During the second wave, the average weekly cases were 7.2 times (95% interval: 6.3—8.2) the average weekly cases of the first wave, but for weekly deaths this ratio was only 2.6 (2.4—2.8). The discrepancy between average weekly normalized cases and deaths was even larger for the third wave: 11.8 (10.4—13.4) and 2.4 (2.3—2.6), respectively.

Although anecdotally we know COVID-19 testing became more widely available during this time, part of these discrepancies can be attributed to the differences in age composition among the waves. The median age of cases during the first wave was substantially higher than that during the second wave (Fig 2b), dropping rapidly from 50 to 34 during May. The median age then stabilized at approximately 40 until late 2020/early 2021, when the older population started to get vaccinated. A similar decrease in the median age of death can be seen right after the first wave from 83 to 75 before stabilizing at around 79.

We can obtain further insights by stratifying the data by age group. Again using the first wave as the baseline, there were disproportionately more cases in the youngest age group (0 to 39) than in the oldest age group (above 65) in the upcoming waves (Fig 2c). Overall, the average normalized cases were 11.2 (9.1—14.3), 6.2 (5.5—7.0), and 3.9 (3.6—4.2) for ages 0 to 39, 40 to 64, and above 65, respectively, during the second wave and 18.3 (15.0—23.1), 10.0 (9.0—11.2), and 6.7 (6.3—7.2) for the third wave.

In contrast, normalized deaths were similar among age groups, except in the beginning of the second wave and the end of the third wave Fig 2d. Average weekly normalized deaths for the second wave were 3.0 (2.2—4.2), 3.0 (2.7—3.3), and 2.5 (2.4—2.7) for 0 to 39, 40 to 64 and above 65; 2.2 (1.5 to 3.1), 2.6 (2.4—2.9), and 2.4 (2.3—2.6) for the third wave. From Jun to Jul 2020, the youngest age group led those above 65 in normalized deaths by at least one week. As there is no reason to believe young people would die faster from COVID-19 than seniors, this suggests that transmission increased in the youngest age group before the older age group. From Feb 2021 onward, the normalized deaths in those above 65 plummeted much faster than in other age groups, likely due to the age-structured vaccine distribution program.

### Differential drop in case-fatality risk among age groups

Although it is not possible to directly calculate the infection detection probability from these data, we can use the case-fatality risk (CFR) as a proxy to understand changes in infection ascertainment. Here, we define a case as a reported infection as the State of Florida did, irrespective of symptoms. We calculate the CFR by case reporting date directly from the linelist. We normalize these numbers based on the mean CFR during the first wave to observe how the ratio has declined over time. Changes in CFR may be due to either changes in the infection fatality ratio (e.g., more effective medical protocols, more severe variants) or differential changes in ascertainment by outcome severity.

**Fig 3:**
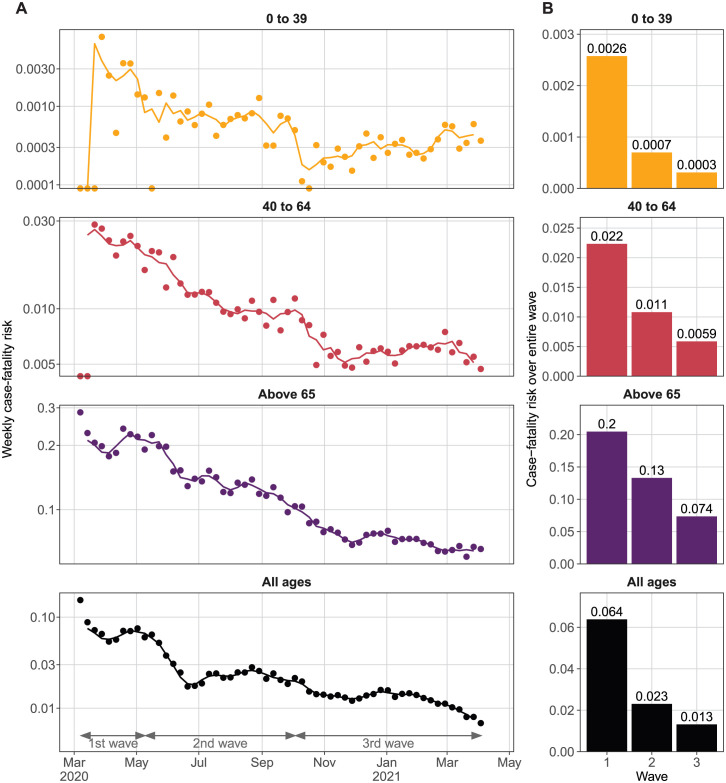
Case-fatality risk (CFR) over the first year of the pandemic. (A) Weekly CFR (dots) and the 3-week moving average (line) for each age group. Note that the y-axis is log-scaled. (B) Average CFR over each wave for each age group.

Changes in all-age CFR occur in three phases that may be related to the transmission waves. After the first wave, the all-age CFR declined rapidly from 6.4% (average of first wave), before stabilizing at around 2.3% from July 2020 until the end of the second wave (Fig 3). The all-age CFR declined again during the third wave, resulting in an average of 1.3% during the third wave. The overall second and third wave CFR were about 1/3 and 1/5 of the first wave CFR, respectively. The CFR continued to decline from Feb 2021 onward, which was when the mass vaccination program was started.

**Fig 4:**
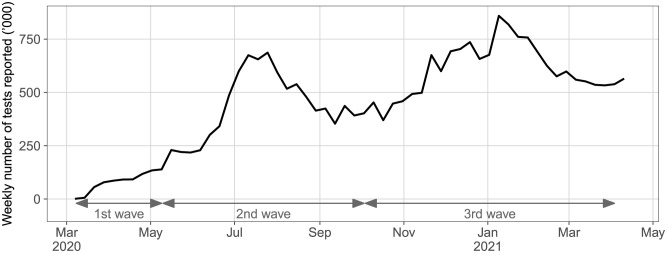
Weekly number of SARS-CoV-2 tests reported by Florida.

The rise in number of tests reported by the state over the same period was faster than the drop in CFR. The mean weekly number of tests increased from 80,000 during the first wave to 438,000 and 593,000 during the second and third waves respectively, or 5.5 and 7.4 times that of the first wave (Fig 4). As expected, the weekly numbers of tests follow the caseload pattern closely.

Although CFR among all age groups followed the same declining pattern as all-age CFR, their rates of decline differed. CFR for 0 to 39 fell immediately after the first wave before stabilizing at around 0.07% for the second wave. Another steep decline for the age group was observed in Oct 2020, and the CFR remained at around 0.03%, which was less than 1/8th of the first wave. Interestingly, CFR appeared to increase again after Feb 2021.

CFR for ages 40 to 64 fell gradually throughout the second wave, reaching less than 1/2 of the first wave CFR at 0.09% by the end of the 2nd wave (Fig 3). Then, the CFR declined rapidly to 0.06% by Nov 2020 and stabilized at this level. The CFR for those above 60 declined the slowest relative to the first wave. The CFR for this age group declined by about 30% between May and the end of Jun 2020, from 0.2 to 0.14. In the third wave, the CFR declined gradually to 0.07 and remained at this level for the rest of the study period.

### Death-to-hospitalization and hospitalization-to-case ratio changed as caseload increased

To investigate whether the ratio among the indicators changed with community transmission, we examined the relationship between caseloads and the case fatality ratio, hospitalization-to-case ratio and death-to-hospitalization ratio. Since hospitalization in the state linelist was unreliable (as described above), we focus here on the HHS hospitalization data. To account for the difference between case report date and hospitalization date, we assumed that the admission date lagged the case reporting date by a week and adjusted the HHS data accordingly to yield hospital admission by the case reporting date. Because HHS data did not capture admission data from all health facilities until Nov 2020, we limited this exercise to the period from Nov 2020 to Apr 2021, which was within the third wave. Since the age demarcations in HHS data were different from that of other datasets including the all-cause mortality, we had to use slightly different age groups for this analysis.

**Fig 5:**
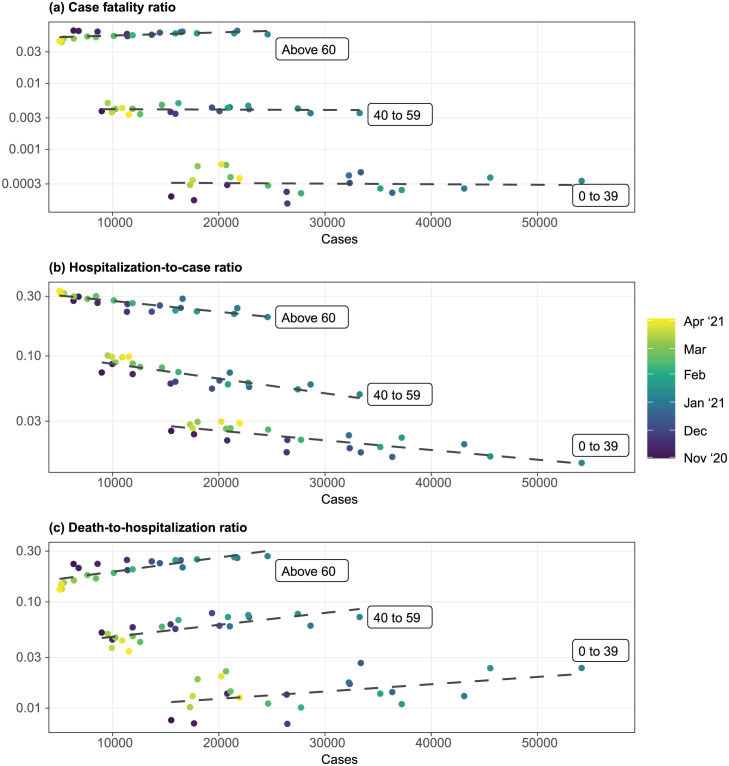
Relationship between weekly case loads and weekly case fatality ratio, hospitalization-to-case ratio and death-to-hospitalization ratio during the third wave. All indicators are indexed using case reporting date. Cases and deaths were derived from the state linelist. Hospitalizations were based on HHS data. We assumed that the admission date lags the case reporting date by a week, and adjusted the hospitalizations time series accordingly. Dashed lines are linear trendlines. Only the third wave is investigated since the first two waves’ hospitalization data was unreliable.

Consistent with the observation from Fig 3, CFR remained steady for all age groups regardless of the caseloads (Fig 5a). Given the assumption that the infection fatality ratio remained similar, this suggests that the case detection probability was consistent and that the use of caseload as an indicator of transmission strength is proper, but only over this period.

As the caseload increased, the hospitalization-to-case ratio decreased (Fig 5b). On the other hand, the death-to-hospitalization ratio increased with caseload (Fig 5c). These trends were consistent among all three age groups.

### Excess deaths were explainable by reported COVID-19 deaths except during summer waves

To understand whether COVID-19 deaths could have been underreported and thus affect our understanding of the CFR, we estimated the weekly excess deaths using all-cause death data provided by CDC. We then compare the excess deaths with reported COVID-19 deaths by date of death.

**Fig 6:**
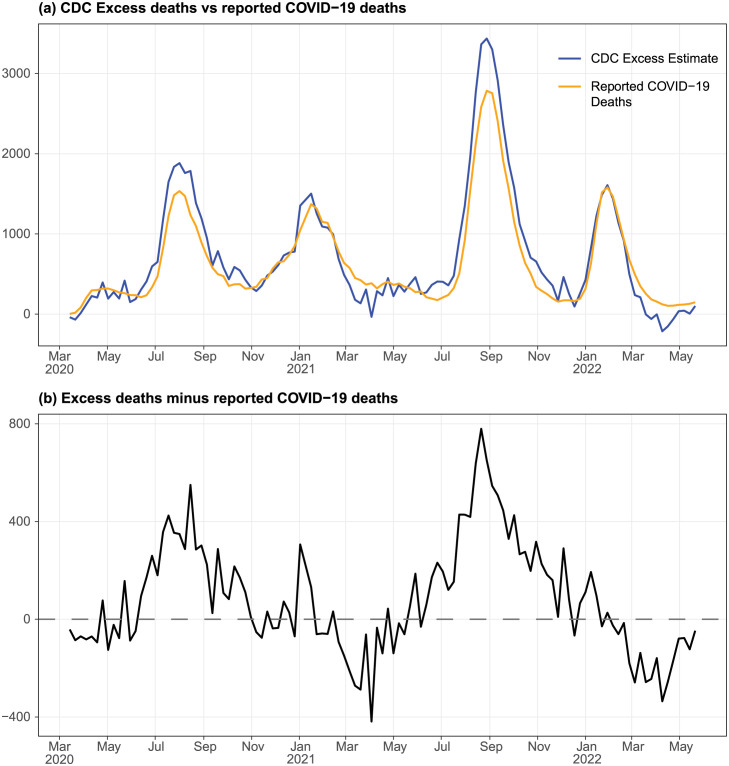
Comparison between excess deaths and reported COVID-19 deaths, from Mar 2020 to May 2022. (a) Excess deaths and reported COVID-19 deaths. We calculate the excess deaths as the observed all-cause deaths minus the expected weekly all-cause deaths based on data from 2015 to Feb 2020. (b) Excess deaths not accounted for by reported COVID-19 deaths.

Excess deaths matched the reported COVID-19 deaths well throughout the pandemic, with some notable deviations (Fig 6a). As many as 800 weekly excess deaths were not accounted for by the reported COVID-19 deaths during the summer waves in 2020 and 2021 (Fig 6b). On the other hand, excess deaths were lower than reported COVID-19 deaths in Mar and Apr in both 2021 and 2022.

## Discussion

The number of cases generally underestimates the actual number of infections in the population, especially during the early days of the pandemic when testing was available only to vulnerable populations and those who experienced severe symptoms. As a result, hospitalization and deaths are more reliable measures for understanding the dynamics of infections, although deaths in particular will be a lagging indicator. Our analyses showed that state hospitalization data during the early months of the pandemic were incomplete, and it is unclear if the incompleteness was entirely random. Thus, reliable hospitalization data was not available for the first eight months of the pandemic, highlighting the importance of understanding case detection dynamics. In contrast, deaths were generally reliably detected, as shown by good matching with the excess deaths except during the summer waves. Because cases and deaths in the state linelist are anchored to a single date (case reporting date), we can derive the case fatality ratio in a straightforward manner and use the trend to understand the changes in the case detection rate based on some assumptions.

The infection detection rate cannot be determined directly, although the CFR can provide some insights. The CFR improved in a stepwise manner with two clear change points coinciding with the beginning of each epidemic wave. In Jun 2020, the CFR was close to 1/3 that of the first wave. This is likely because tests had become increasingly available, especially to younger age groups with an expansion of testing criteria. In October 2020, the CFR improved further, potentially due to the increasing use of routine asymptomatic testing in colleges and workplaces. Younger populations benefited more from these testing practices, as supported by the more drastic changes in CFR. The continued drop in CFR after Feb 2021 is likely due to the fall in the population-wide infection fatality ratio as the older population became vaccinated. Interestingly, the younger population (0 to 39) recorded an uptick in CFR during this period. It is unclear if this was predominantly due to a reduction in detection or an increase in disease severity.

As the availability of testing changed dramatically during the first year of the pandemic, much of the observed trends in CFR were likely due to changes in infection detection. However, improved care, such as the introduction of more effective treatments [17], likely improved the infection- and case-fatality risk. In Jun 2020, a preprint describing the benefit of dexamethasone was released (now peer-reviewed as [18]), coinciding with the drastic decline in CFR, especially among people above age 65. As a result, using changes in CFR to directly infer the changes in infection detection will likely overestimate ascertainment of infections. Nevertheless, this allows us to infer the upper limit of the changes to infection detection. For example, the infection detection in the second wave would have at most increased by 2.8-fold compared to the first wave, though the average weekly number of tests increased by 5.5-fold.

At the time the second wave was occurring, we observed that young people seemed to be accounting for a large proportion of reported cases. A critical question was whether we might be going into a younger and thus less severe wave, or if transmission would spill over into senior populations, driving an increase in deaths. Here we show that both were at play: CFR dropped, causing the wave to be less severe than the case numbers at the time suggested, but normalized deaths suggest that transmission actually did peak 1 to 2 weeks earlier in the younger population than the older population. After this, normalized deaths remained similar among all age groups for the rest of the study period, until the age-structured vaccination program started. The phenomenon of younger people driving early transmission during a wave has been observed elsewhere ([19]), and has a likely explanation in contact network theory: individuals with more interactions (often students and workers, in this case) tend to get infected before those with fewer (often retirees) ([20]).

The pairwise ratios among cases, hospitalizations, and deaths suggest that there were fine-scale variations in the case detection rate and infection fatality ratio within a single wave that were hard to disentangle using publicly available data. During the third wave, there were more deaths per hospitalization as caseloads increased but fewer hospitalizations per detected case, resulting in a stabilized CFR across all age groups. Two hypotheses could explain these patterns. First, hospitals might have admitted only more severe cases as cases surged, resulting in disproportionately more deaths among those hospitalized and fewer hospitalizations among diagnosed cases. It is unclear if *de facto* hospital admission criteria were changed during the peak of the wave to support this hypothesis, although to our knowledge formal admission criteria did not change. It is possible that hospital policy did not change, but healthcare-seeking behavior did, e.g. as emergent care wait times increased. Alternatively, the quality of care might have decreased as more people were hospitalized, reducing the ratio of intensive care specialists to patients and thus an increase in deaths among hospitalized patients. Meanwhile, case detection for mild symptomatic or asymptomatic infection at the peak of the wave might have improved as people were more conscious about ongoing community transmission. The infection fatality ratio and case detection rate would have to increase and work in tandem to stabilize the CFR as transmission intensified under this hypothesis.

Underreporting of COVID-19 deaths affects some of our analyses. Most excess deaths unaccounted for by reported COVID-19 deaths occurred during the height of summer waves in 2020 and 2021. This could be due to underreporting of COVID-19 deaths as the surveillance systems were overwhelmed, or that care for non-COVID-19 illness diminished with COVID-19 surges. The former would have impacted our analysis of the case detection rate during the second wave since the actual CFR might have been higher resulting in a lower case detection rate than our estimates. Nevertheless, this would have no impact on our estimates for the third wave and our analysis of the pairwise case, hospitalization and death ratios, which was based only on the third wave.

Much of our analysis would not be possible without a publicly available linelist of reported cases. Linelist data has been crucial in understanding heterogeneity in transmission trends, risk, and disease burden (e.g. [19, 21–24]) and in parameterizing mathematical transmission models ([25, 26]). In the United States, few states provided publicly available or daily updated linelist data as Florida did, let alone both. Unfortunately, this extremely valuable resource was discontinued in early Jun 2021, preventing us from assessing how case reporting rates changed as vaccination and at-home testing became common. Nevertheless, the linelist data from the first 16 months of the pandemic is key to better understanding and interpreting the case, hospitalization, and death dynamics during the early pandemic period when the detection rate was likely the most variable. As a result, it enables the parameterization and development of transmission models that properly recreate the transmission pattern in Florida ([27, 28]). Making linelist data readily available will help lower the barrier for researchers to learn more from COVID-19 as we prepare ourselves to respond more effectively to future public health emergencies.
